# Assessing Functional Connectivity Dynamics During Cognitive Tasks Involving the Dorsal Stream

**DOI:** 10.3390/e27060566

**Published:** 2025-05-27

**Authors:** Huifang E. Wang, Jorge Gonzalez-Martinez, Viktor Jirsa, Patrick Chauvel, F.-Xavier Alario, Catherine Liegeois-Chauvel

**Affiliations:** 1 Faculté de Medécine, Institut de Neurosciences des Systèmes (INS), Aix-Marseille Université, Institut National de la Santé et de la Recherche Médicale (INSERM), 13005 Marseille, France; viktor.jirsa@univ-amu.fr; 2 Department of Neurological Surgery, University of Pittsburgh, Pittsburgh, PA 15260, USA; gonzalezjo@upmc.edu; 3 Epilepsy Department, Neurological Institute, Cleveland Clinic, Cleveland, OH 44195, USA; chauvep2@ccf.org; 4 Centre de Recherche en Psychologie et Neurosciences (CRPN), Centre National de la Rechereche Scientifique (CNRS), Aix-Marseille Univerité, 13003 Marseille, France; francois-xavier.alario@univ-amu.fr

**Keywords:** functional connectivity, cognitive tasks, dorsal language stream, connectivity profiles

## Abstract

Functional connectivity and its dynamic reconfiguration during cognitive tasks offer valuable insights into the neural mechanisms underlying cognitive functions. The dorsal language stream plays a crucial role in linking auditory and visual information with motor functions during language-related tasks. In this study, we investigated the dynamic functional connectivity of brain regions within the dorsal stream across five cognitive tasks using invasive stereoelectroencephalography (SEEG) recordings from patients with drug-resistant epilepsy. Our results reveal distinguishable functional connectivity patterns across various cognitive tasks using clustering algorithms. Furthermore, we were able to identify specific cognitive tasks based on their unique functional connectivity signatures, with a median of accuracy 0.91. Additionally, we identified key brain regions with strong connectivity roles and high variability across tasks. We analyzed source (out-degree) and sink (in-degree) regions during the picture naming, ba/pa, and oddball tasks, highlighting both shared and task-specific connectivity patterns. Among the twenty or so brain regions displaying a median in- and out-degree > 0.5 during the three tasks, the middle frontal gyrus (MFG) was highly involved in all three, corroborating its critical role in cognition. In contrast, the left superior frontal gyrus (SFG) and the superior temporal gyrus appeared to be modulated specifically via the tasks, exhibiting greater activity during picture naming compared to the other tasks. These findings enhance our understanding of the dynamic connectivity profiles associated with cognitive processing within the dorsal stream.

## 1. Introduction

Functional connectivity describes how the brain coordinates activity across different regions by quantifying the communication and synchronization of neural signals [1]. The mechanisms underlying functional connectivity involve complex interactions at multiple levels, including neural synchronization, long-range connections, oscillatory activity, network interactions, and neuroplasticity. Task-based functional connectivity reveals how brain networks dynamically reconfigure to support specific cognitive demands, with certain connections emerging or strengthening in response to task-related processing. This allows researchers to distinguish functionally meaningful connectivity from incidental co-activation [2]. Furthermore, dynamic functional connectivity—tracking changes in connectivity over time—provides insight into the brain’s adaptive and fluctuating states, offering a richer understanding of ongoing cognitive processes [3].

The dorsal streams in both vision and language represent pathways that emphasize sensorimotor integration and the real-time guidance of actions, though they operate in different modalities. Both dorsal streams manage real-time, action-related transformations—the visual dorsal stream is involved in eye–hand coordination and the visual control of action, primarily described in primates and later studied in humans (for a review, see [4]). The language dorsal stream, in contrast, supports the mapping of sound to articulation [5]. A common theme between the two is the transformation of perception into action, though in different domains.

The dorsal stream (DS) is one of the two main neural pathways involved in language processing, along with the ventral stream [6,7,8]. The parieto-frontal network of DS primarily supports phonological processing, speech production, and auditory–motor integration. It maps sounds to articulation, linking auditory representations of speech with motor commands for overt production. The DS is also critical for organizing words in sentences. The inferior and middle frontal gyri (IFG and MFG), along with the pre-SMA, are involved in word selection processes, particularly when selecting among object names and action verbs [9,10,11,12]. The anatomical pathways of the DS include the arcuate fasciculus and the superior longitudinal fasciculus, which connect the Sylvian–parieto–temporal (SPT) region to the inferior frontal gyrus (IFG) and the premotor cortex [6]. The SPT region is a junction area that comprises the superior temporal gyrus (STG), the superior temporal sulcus (STS), and the posterior Sylvian fissure at the intersection of the posterior temporal, angular, and inferior supramarginal gyri. In a previous study using SEEG, we identified the cortical structures known to be involved in word production (for a review of fMRI evidence, see [13]), focusing on the parieto-frontal network referred to as the dorsal stream pathways [14]. These identified brain regions are used here to guide functional network studies.

In the present study, we started by characterizing the brain functional network recruited via a picture naming task. Because many of the regions involved are also engaged during non-linguistic cognitive tasks, including auditory discrimination, visual divided attention, and spatial problem solving (for a review, see [15]), we expanded our investigation to include additional tasks to assess the functional specificity and shared network nodes. For example, passive listening to syllables (e.g., the ba/pa task) is supported by a temporo-parieto-frontal network that, in addition to auditory areas, involves the inferior parietal gyrus, the middle frontal gyrus, and the pars opercularis of the IFG [16]. The oddball task elicits a P300—a positive component of the event-related potential associated with processes such as encoding, identification, and categorization, and generated via a widely distributed network including fronto-parietal areas [17,18]. Thus, we compared the functional networks elicited via a perceptual auditory language task (picture naming), passive listening to syllables (ba/pa), word/non-word discrimination, and two visual tasks: the oddball task and face recognition. This comparison aims to identify common network nodes and better understand the directionality of connections (forward vs. backward) across tasks.

A fronto-parietal domain-general network, the multiple-demand (MD) system, is thought to support a broad range of cognitive functions across diverse tasks [19,20]. As noted by Fedorenko et al. (2013) [20], most of the supporting data come from group-level analyses, which may overestimate activation overlap due to inter-individual variability. More precise conclusions regarding MD region activations emerge when analyses are performed at the level of individual subjects. For example, within Broca’s area, it becomes possible to distinguish a language-selective subregion from a neighboring domain-general region, which are anatomically adjacent but functionally distinct [21]. The reliability of such differentiation improves further when intracerebral recordings are used; they offer both high spatial and temporal resolution. Using this approach, Liégeois-Chauvel et al. [14] investigated inter-individual variability in dorsal stream dynamics during speech production and demonstrated within-region heterogeneity in activation patterns across the fronto-parietal language network. In our approach, we also account for inter-individual variability to ensure more accurate and personalized mapping of functional networks.

Historically, techniques such as scalp electroencephalography (EEG) and functional magnetic resonance imaging (fMRI) have provided valuable insights into functional connectivity [2,22]. We selected the invasive stereo-electroencephalography (SEEG) technique, commonly used for epilepsy patients, which enables the investigation of deeper and more specific brain regions with high temporal resolution. SEEG signals offer precise anatomical localization and capture the temporal dynamics of neuronal populations at the millisecond scale. Because electrodes are implanted directly into the brain, SEEG can reveal functional interactions both within and between regions, with multiple nodes monitored simultaneously [23,24,25]. Another invasive intracranial recording method, electrocorticography (ECoG), also used for epilepsy patients, offers high spatial resolution but is limited to the brain’s surface. The spatial resolution of ECoG typically ranges from 100 to 500 µm, compared to SEEG, which ranges from 2 to 5 mm. However, ECoG is generally restricted to the cortical surface and covers a few of brain regions, while SEEG can access both the cortical surface and deep brain structures, offering broader coverage and including deep areas such as the hippocampus, the insula, and the thalamus. Most functional connectivity studies using SEEG and ECoG focus primarily on the diagnosis of epileptogenic zones and functional mapping [26,27,28]. For task-related studies, the focus is more on understanding the roles of brain regions in performing specific cognitive tasks [14,23,29].

Various methods are used to measure functional connectivity, each differing in approach, sensitivity, and temporal or spatial resolution. In this paper, we applied the Multiple Connectivity Analysis algorithm (MULAN: [30]) to assess and contrast directed functional connectivity in SEEG recordings during different cognitive tasks. MULAN employs an ensemble-based approach that combines multiple analysis methods with fuzzy logic to extract the most probable connectivity structures from neuronal networks. In this study, we selected a combination of information, including correlation, network delay information, directionality, model-based coherence, and cross-spectral information.

The main contributions of this work are as follows. (1) Task-specific connectivity fingerprints: we found that each cognitive task exhibited a unique connectivity pattern, enabling high-accuracy task classification (median accuracy = 0.91), which highlights the discriminative power of dynamic functional connectivity. (2) The identification of key hub regions: our analysis revealed brain regions with strong and variable connectivity roles, offering insights into their task-specific and domain-general functions. (3) The directionality of information flow: by examining source (out-degree) and sink (in-degree) dynamics during picture naming, ba/pa, and oddball tasks, we shed light on how information flows through the network across task types. (4) Reproducible analysis workflow: we provide a detailed pipeline for investigating dynamic directed functional connectivity from SEEG data, which can be adapted for future studies.

## 2. Materials and Methods

### 2.1. Patients

Epileptic patients were recruited from the pool of patients assessed at the Cleveland Clinic Epilepsy Center for surgical treatment of medically refractory epilepsy. They were undergoing a stereo-electro-encephalography (SEEG) diagnostic evaluation as part of their pre-surgical assessment. In brief terms, the SEEG methodology involves the rigorous scrutiny of all the evidence obtained during the noninvasive phase of pre-surgical evaluation to define a coherent hypothesis regarding the likely localization of the epileptogenic zone and its propagation network [31,32]. After an anatomo-functional localizing hypothesis is formulated, a strategy to implant depth electrodes to specific regions of the brain is defined. Depth electrodes are stereotactically inserted using a robotic surgical implantation platform (ROSA, ROSA One, Brain MEDTECH S.A., Mauguio, France), in a three-dimensional arrangement with either orthogonal or oblique orientations, allowing intracranial recordings from lateral, intermediate, or deep cortical and subcortical structures [33,34]. For each patient, between 8 and 13 stereotactically placed electrodes were implanted. Each electrode contained 8–15 recording channels, which were 2 mm long, 0.8 mm in diameter, and spaced 1.5 mm apart. The insertion of these electrodes is based purely on clinical needs and is made independently of any research-related purpose [35].

The current study involved 12 epileptic patients who were native speakers of English. Table A1 provides detailed demographic and clinical information for each patient. The study included a total of 12 participants (7 female, 5 male), aged between 21 and 61 years (M = 40.1, SD = 11.8). Handedness was predominantly right-handed (10 right-handed, 2 left-handed), and language dominance was primarily left-hemispheric, assessed via clinical criteria (ictal aphasia, Wada testing, fMRI, and electrical stimulation). Participants presented with drug-resistant focal epilepsy with various foci, including mesial temporal, lateral temporal, and temporo-parietal regions. Patients were invited to participate in a picture-naming experiment when their implantation included depth electrodes within the parieto-frontal networks known as the dorsal language stream [36]. Eight patients participated in ba/pa tasks, five patients completed the oddball experiment, two patients took part in the face recognition task, and one patient was involved in the words vs. non-words discrimination task. All patients enrolled voluntarily after giving written informed consent under criteria approved by the Cleveland Clinic Institutional Review Board (N°13-1248). Demographic and clinical data are summarized in Table A1.

### 2.2. Anatomical Reconstruction of Electrode Positions

MRI acquisition. All MRI scans were acquired from a 3T Siemens Skyra scanner (Siemens, Erlangen, Germany) at the Cleveland Clinic Foundation. The T1-weighed Magnetization Prepared Rapid Acquisition with Gradient Echo (MPRAGE) volumetric scan was used for co-registration with CT. MRI and CT fusion. Immediately after SEEG implantation, high-resolution stereo-computerized tomography (CT) was taken to obtain the anatomical location of the implanted electrodes. The post-implantation CT scan was exported into Curry 7 (Compumedics Neuroscan, Hamburg, Germany), and co-registration with the MPRAGE images was performed using an automated full-volume registration with maximization of mutual information. The accuracy of the co-registration was inspected visually and confirmed in all patients by a neurosurgeon (JGM) to ensure accuracy. The location of each electrode contact, determined by the center of the highest intensity on the CT, was individually labeled and superimposed on the MRI for visualization of its anatomical location. Normalization of MRI and CT. Further processing was performed within SPM8 (Wellcome Department of Cognitive Neurology, London, UK) in MATLAB 2015a (MathWorks, Natick, MA, USA), in order to normalize the locations of the electrodes of interest from all the patients. Three processing steps were performed: (1) co-registration of the CT to the MRI for each individual patient; (2) normalization of the individual MRI with the Montreal Neurological Institute (MNI) 3D common stereotactic space [37]; and (3) normalization of the co-registered CT in step 1 to the MNI space by applying the same transformation matrix as obtained in step 2. All the normalized CT and MRI images were then fused in the Curry 7 software (Compumedics-Neuroscan, Singen, Germany) so that the electrodes could all be superimposed on the normalized MRI and visualized on a template according to the Talairach stereotactic coordinate system. Given the focus of this research, our exploration was limited to regions located within the language dorsal stream (see Section 1) and organized into seven anatomical groups. Each contact (n=447) was localized to a specific brain region of the Talairach atlas [38] based on its coordinates and confirmed by individual visual verification.

### 2.3. SEEG Data Acquisition

SEEG electrophysiological data were acquired using a clinical electrophysiology recording system (Nihon Kohden 1200, Nihon Kohden America, Irvine, CA, USA) at a sampling rate of 1000 Hz. During the cognitive tasks, stimulus and behavioral event data were simultaneously recorded along with the SEEG signals [39] and stored for subsequent analysis. All recordings were referenced online to a scalp electrode placed at Fz.

### 2.4. Cognitive Tasks

#### 2.4.1. Picture-Naming Experiments

Patients were asked to name out loud pictures of common objects from the Snodgrass and Vanderwart (1980) [40] collection. There were 36 common items, chosen from six different semantic categories (accessories, buildings, kitchen utensils, fruits, furniture, and musical instruments). During a preliminary familiarization phase, the patients named these items once and were provided corrective feedback in cases where unexpected responses were given. Then, the experimental task proper started. The pictures were presented in short blocks each involving six items repeated five times, yielding 30 trials per block. A total of 8 blocks was planned for each patient (i.e., 240 trials), but not all patients completed all the blocks due to clinical circumstances (e.g., fatigue or interruptions). (See the details of the design in Liegeois-Chauvel et al., 2022 [14]). The experiment was controlled via the software E-Prime v2.0.1 (Psychology Software Tools, Pittsburgh, PA, USA). The pictures were presented on the center of the screen within a visual angle of 6° × 6°. A trial consisted of a fixation point (variable duration across trials, between 1400 and 2100 ms), followed by the black and white target picture (presented for a fixed duration of 1000 ms). Various pseudo-random orders were created to vary across patients the order of items within a block and the order of the blocks within the experiment.

#### 2.4.2. Oddball Experiments

The visual oddball task (OB) consisted of 250 trials (20% targets), the duration of the presentation of each stimulus was 400 ms, and the interstimulus interval (ISI) varied between 1000 and 1600 ms. The target and distractor were the same throughout the task and were abstract pictures difficult to verbalize. The patients had to mentally count the rare, target stimuli, which appeared randomly among distractors (frequent). They reported the number of targets they had counted after the end of the task.

#### 2.4.3. ba/pa English/French

Four *syllables*: /ba/ and /pa/ pronounced by an English or French female were presented through headphones in a pseudo-randomized order at a 22 kHz rate using E-prime 1.1 (Psychology Software Tools Inc., Pittsburgh, PA, USA) 500 times in total with an ISI of 1030 (±200) ms. The patients were instructed to listen passively and concentrate on what they heard.

#### 2.4.4. Face Recognition

Patients viewed 48 faces of celebrities in gray-scale and 48 faces of unknown people displayed on a screen for 396 ms with an ISI between 1300 and 2000 ms. They had to say, for each, whether they knew the face or not. This procedure was not intended to assess how the patients recognized the faces (e.g., by familiarity or naming).

#### 2.4.5. Word vs. Non-Word (W/NW) Discrimination

Patients listened to consonant–vowel syllables and identified those which were valid words.

### 2.5. Functional Connectivity and Its Dynamics

We calculated functional connectivity using a sliding-window approach with a 5 s period and 50% overlay. The selection of a 5 s window was based on a systematic evaluation of functional connectivity algorithms for generating meaningful connectivity matrices from EEG/SEEG signals [41]. We used the MULAN algorithm [30] to estimate directed and weighted functional connectivity from the SEEG signals. MULAN is a robust ensemble method that combines multiple connectivity measures to extract the most probable connectivity structures in complex neuronal systems. In this study, we selected four comments used functional connectivity methods, BCorrU, COH1, BCorrD, and PDC, utilizing fuzzy logic to synthesize their outputs and enhance reliability. To optimize the integration parameters of the MULAN algorithm, we used a genetic algorithm. This evolutionary optimization process was applied to simulated SEEG-like signals generated from networks with 30 nodes. The genetic algorithm allowed us to identify a suitable distribution of fusion weights for each connectivity measure under realistic signal conditions, ensuring that MULAN could generalize well to real patient data.

Among the four measures included in MULAN here, BCorrU is a simple bivariate method that computes pairwise Pearson correlation coefficients between signals. It captures undirected, linear associations. In contrast, BCorrD introduces a directional component to correlation-based analysis. It compares the predictive power of the past activity of node *i* on the present activity of node *j* against the reverse, and it infers a direction of interaction accordingly—i.e., from *i* to *j* if the past of *i* better predicts the current state of *j* than vice versa. The other two metrics, COH1 and PDC, belong to the so-called $\bar{A}H$ family of methods [41]. These are model-based approaches that estimate directed interactions by fitting a multivariate autoregressive (MVAR) model to the time series data. The connectivity is analyzed in the frequency domain using the matrix of MVAR coefficients A(f); *f* refers to a frequency. Partial directed coherence (PDC), originally introduced by Baccalá and Sameshima [42], is a normalized form of A(f) designed to quantify the strength and directionality of interactions relative to a given source. It evaluates the influence of past activity from one signal ($x_{j}$) over another signal ($x_{i}$), normalized across all potential targets, which allows a ranking of directional influences. On the other hand, COH1 is derived from the cross-spectral power density matrix, which is, in turn, calculated from the inverse of the transfer function $\bar{H}\left(f\right)=\bar{A}{\left(f\right)}^{-1}$ and the covariance matrix of the residuals. This measure accounts for prediction errors and provides a normalized coherence value between signal pairs in the frequency domain.

The elements of the functional connectivity dynamics matrix are the Pearson correlations between two functional connectivities from two different sliding windows.

### 2.6. Clustering Methods for Distinguishable Functional Connectivity

In this study, we first pooled all functional connectivity obtained during different tasks. To automatically group these functional connectivity patterns, we selected two cluster algorithms, spectral clustering and BIRCH clustering, based on their superior performance compared to 9 other algorithms available in the scikit-learn 1.6 package of Python [43]. Spectral clustering [44] initially constructs a similarity matrix based on the functional connectivities. Next, the Laplacian matrix is computed from this similarity matrix, followed by eigenvalue decomposition to compute its eignevectors and eigenvalues. The clustering is then determined based on the properties of these eigenvectors. One advantage of spectral clustering is that it makes no assumptions on the shapes of clusters, allowing it to handle complex geometric structures effectively.

BIRCH clustering [45], which stands for Balanced Iterative Reducing and Clustering using Hierarchies, is a hierarchical clustering algorithm. It builds a tree-like structure, called a Clustering Feature Tree, to represent the data distribution. The tree is constructed recursively by merging similar clusters until meeting a stopping criterion, such as reaching the maximum number of sub-clusters or the maximum diameter of a cluster. BIRCH is known for its ability to detect clusters of arbitrary shapes and sizes.

To explore the underlying structure of the functional connectivity during different tasks in a visually interpretable way, we applied t-distributed stochastic neighbor embedding (t-SNE), a nonlinear dimensionality reduction technique that preserves local relationships and is particularly effective for visualizing high-dimensional data in two or three dimensions. This method helps reveal a latent structure that may not be apparent in the original feature space [46].

### 2.7. Machine Learning Methods for Identifiable Tasks

We selected two machine learning methods to identify cognitive tasks from a given functional connectivity. To obtain the training and test set of functional connectivity, we used stratified K-fold cross-validation as implemented in scikit-learn [43] to ensure a balanced distribution of tasks across folds. In each fold, 80% of the data was used for training and 20% for testing. The data were separated by time to guarantee that there was no overlap between the training and testing data. Given a functional connectivity matrix, we used the machine learning methods to identify tasks. The two selected machine learning methods were a random forest classifier (RFC) and a support vector machine (SVM).

RFC [47] is a robust machine learning algorithm that reduces overfitting by constructing multiple decision trees during training and outputting the mode of the individual trees. RFC randomly selects a subset of the training data (with replacement) to train each decision tree. At each node of the decision tree, RFC considers only a random subset of the features to make a split, reducing the correlation between trees and improving diversity. Each decision tree is grown to its maximum depth or until a minimum number of samples is reached in each leaf node. The class predicted via each decision tree is considered, and the most common class is chosen as the final prediction.

SVM [48] is a powerful supervised learning algorithm that identifies the optimal hyperplane to separate classes in feature space. This hyperplane maximizes the margin, which represents the distance between the hyperplane and the nearest data points from each class, also referred to as support vectors. In our study, we utilized SVM to predict functional connectivity data, aiming to minimize the deviation between predicted values and true target values while simultaneously maximizing the margin.

### 2.8. Performance Metrics

We used seven metrics in a Python package named skicit learn [43]. Normalized mutual information (MI): MI(Y,T)=2×I(Y,T)/(H(Y)+H(T)), where *Y* and *T* are predicted values and true values. H(.) is entropy, and I(Y,T)=H(Y)−H(Y|T) is mutual information between *Y* and *C*.

Where TP is the number of true positives (i.e., the number of pair of points that belongs in the same clusters in both *Y* and *T*), FP is the number of false positives (i.e., the number of pair of points that belong in the same clusters in *T* and not in *Y*), and FN is the number of false negatives (i.e., the number of pair of points that belongs in the same clusters in *Y* and not in *T*). The Fowlkes–Mallows index (FM) [49] is defined as the geometric mean between of the precision and recall: FM=TP/sqrt((TP+FP)∗(TP+FN)). A high value indicates good similarity between two clusters.

Accuracy and the Rand index compute a similarity measure between two clusters by considering all pairs of samples and counting pairs that are assigned in the same or different clusters: RI=ACC=(TP+TN)/(TP+FP+FN+TN). The adjusted Rand (AR) is, in essence, the RI adjusted for chance. Balanced accuracy score (BAS) =1/2(TP/(TP+FP)+TN/(FN+PN)). The positive predictive value or precision is TP/(TP+FP).

The diagonal of the confusion matrix with three labels represents the true positive predictions. Each column corresponds to the false negative predictions for the respective row label. (The ba/pa is identified during other tasks).

The Hamming loss computes the average Hamming distance between *Y* and *T*. 0 is for Y=T and a greater Hamming loss, the bigger difference between *Y* and *T*. The mean absolute error (mae) function computes the mean absolute error between *Y* and *T*.

All evaluation metrics in the learning approach were computed using the aggregated method—by concatenating all predictions and ground truths across the five folds. In our cases, the resulting values were very similar to those obtained by computing the mean of individual metrics from each fold (i.e., per-fold evaluation).

### 2.9. Network Profile Metrics

Centrality indices characterize important nodes, but the term “importance” encompasses various meanings, resulting in diverse centrality definitions. In this study, we selected six metrics.

Degree centrality defines the number of links tied to a node. Given the directional nature of our functional connectivity, we considered both in-degree and out-degree. The in-degree of a node is normalized according to its incoming links, while the out-degree is normalized according to its outgoing links.

The closeness centrality of a node measures the average length of the shortest path between the node and all other nodes in the graph. Consequently, the more central a node is, the closer it is to all other nodes. This centrality metric is calculated using the formula

$$c_{C}\left(v\right)=\frac{n-1}{\sum_{u\in V}d\left(u,v\right)},$$

where *V* is the set of nodes, and d(u,v) denotes the distance of the shortest path between *v* and *u* [50]. In this study, we considered both the incoming and outgoing distance to node *v*.

Betweenness centrality quantifies the number of times a node lies on the shortest path between two other nodes. Assuming that communication between brain regions tends to minimize the number of intermediary regions, the node with the highest betweenness facilitates the most communication. The betweenness centrality of a node *v* is computed as the sum of the fraction of all pairs’ shortest paths passing through this node, as described in the formula

$$c_{B}\left(v\right)=\sum_{s,t\in V}\frac{\sigma (s,t|v)}{\sigma (s,t)},$$

where *V* represents the set of nodes, σ(s,t) denotes the number of shortest (s,t)-paths, and σ(s,t|v) is the number of those paths passing through some node *v* other than s,t [51].

Katz centrality, introduced by ([52]), computes a node’s centrality based on the centrality of its neighbors. It is a generalization of the eigenvector centrality. The Katz centrality for node *v* is calculated as follows:

$$x_{v}=/alpha\sum_{u=1}^{N}A_{vu}x_{u}+\beta ,$$

where *A* is the functional connectivity matrix. The parameter β controls the initial centrality and $\alpha <(\lambda_{max}^{-1})$. We calculated both in-edges’ and out-edges’ Katz centrality.

## 3. Results

### 3.1. Overview of the Analysis and Results

**Figure 1:**
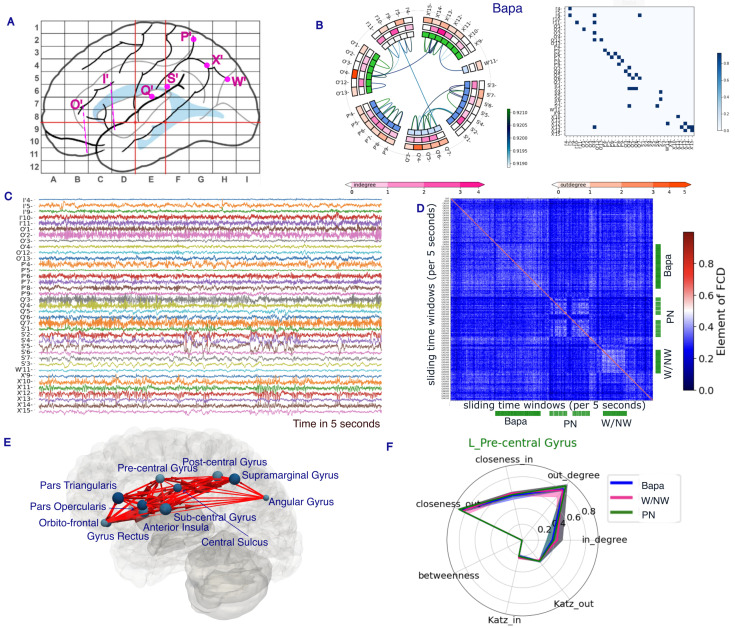
Overview of the analysis using one patient example. (**A**) SEEG implantation scheme. (**B**) Functional connectivity during the Bapa task at the electrode level. (**C**) Selected SEEG time series over a 5 s period. (**D**) Functional connectivity dynamics, with each element representing the similarity of two functional connectivity on different 5 s periods. The green bars indicate when the corresponding tasks were preformed. (**E**) The average functional connectivity with diction and weights during the Bapa task at the brain region level. The size of the circles represents the in-degree of nodes with this network. (**F**) The network profile for a given brain region, defined by in-degree, out-degree, closeness-in, closeness-out, betweenness, and eigencentrality. The different colors represent different tasks; the solid lines and ranges in the profile chart show the mean and percentile (25–75%), respectively.

We analyzed directed functional connectivity in 12 patients, each of whom completed at least two cognitive tasks. To illustrate our analysis workflow, we demonstrated results from a representative patient in Figure 1. Functional connectivity was computed using SEEG time-series data (Figure 1C) within a 5 s sliding window and a 2.5 s overlap. The selected SEEG electrodes were located in dorsal stream regions relevant to language processing (Figure 1A). Directed functional connectivity was estimated using the MULAN algorithm [30], an ensemble-based method that combines multiple connectivity measures with fuzzy logic integration. Connectivity was first computed at the level of bipolar electrodes (Figure 1D). We then analyzed the dynamics of functional connectivity over time by computing pairwise similarities between connectivity matrices from different sliding windows. In this example patient, we observed visually distinct connectivity patterns corresponding to the ba/pa, picture naming, and word vs. non-word (W/NW) tasks. These patterns suggest a task-specific reconfiguration of functional networks. Furthermore, we evaluated whether functional connectivity profiles could reliably distinguish between different tasks. Finally, we used simple classification models to identify the cognitive task based on a given functional connectivity snapshot.

In addition, we performed region-level analyses to examine network profiles at both the individual and group levels. To define connections between brain regions, we selected the maximum weight among all links connecting electrodes within each pair of regions (as illustrated in Figure 1E). Subsequently, we computed six network measures for each brain region to characterize its network profile. We illustrated the variations in network profiles across different cognitive tasks (as depicted in Figure 1F). Finally, we provided the network profiles for all brain regions involved across 12 patients.

### 3.2. Distinguishable Functional Connectivity During Different Cognitive Tasks

**Figure 2:**
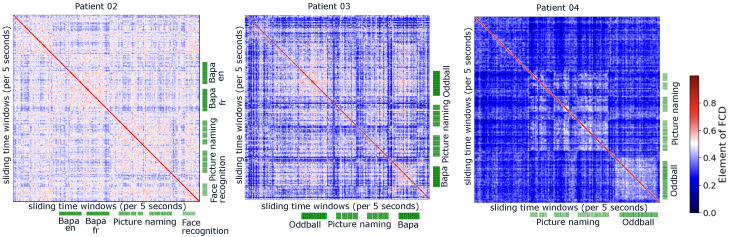
Functional connectivity dynamics matrix of three patients. Each element in the matrix represents the similarity of two functional connectivities during different 5 s periods. The green bars indicate the timing of corresponding tasks.

Is functional connectivity distinguishable during different cognitive tasks? The functional connectivity dynamics have demonstrated that we can visually observe distinct blocks when patients perform various cognitive tasks. In the case of the example patient, the similarity of functional connectivity of 37 electrode channels from 7 electrodes formed the three blocks corresponding to the periods when patients performed three tasks: ba/pa, picture naming, and W/NW tasks (see Figure 1D). We further examined functional connectivity dynamics of three other patients during different tasks, each involving a different number of electrodes (see Figure 2). This led to the question of whether we can distinguish these functional connectivity into distinct clusters corresponding to different tasks.

**Figure 3:**
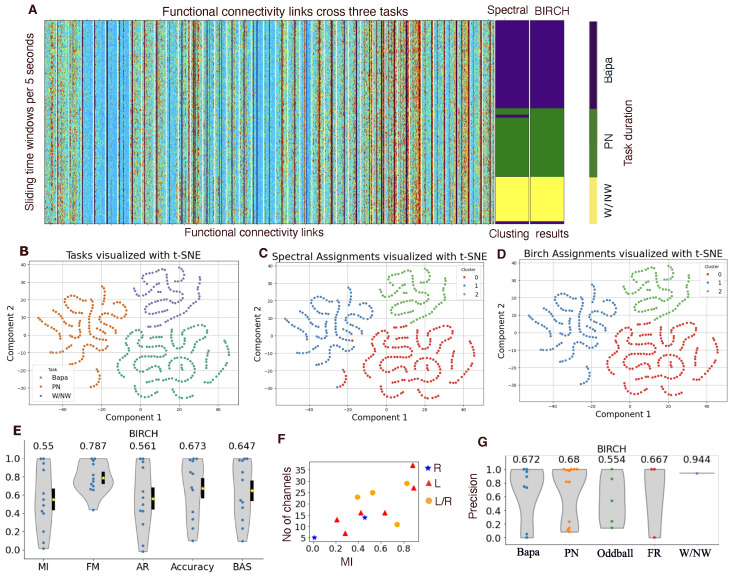
Distinguishable functional connectivity during different cognitive tasks. (**A**) left: the functional connectivity links between electrode leads (x-axis) and sliding time windows (y-axis). Middle: the cluster results from two algorithms, spectral and BIRCH clustering. Right: the time windows during different tasks, ba/pa, picture naming (PN), and W/NW. These results are from the same patient as Figure 1. (**B**) Functional connectivity projected into 2D t-SNE space, with colors indicating the three tasks. (**C**,**D**) Functional connectivity projected into 2D t-SNE space with with colors representing clusters identified via the spectral (**C**) and BIRCH (**D**) clustering algorithms. In (**B**–**D**), each dot responses the functional connectivity during a different sliding window, corresponding to a row on the left of (**A**). (**E**) Performance of BIRCH clustering to group the functional connectivities during different tasks through five metrics: normalized mutual info (MI), Fowlkes–Mallows (FM), adjusted Rand (AR), accuracy, and balanced accuracy score (BAS). Violin plot in gray and dots for patients. The yellow stars are mean values, and noted in the top of each violin plot and the black bar is from the 25th to 75th percentiles. (**F**) Number of electrode channels in a function of MI. The blue stars represent the patients with electrodes implanted only in the right hemisphere, and the red triangles represent those with electrodes only in the left hemisphere, while the orange dots represent those with electrodes implanted in both hemispheres. (**G**) Precision for BIRCH clustering in terms of different tasks.

For the example patient, we applied two clustering algorithms to partition these functional connectivities into three groups, with each corresponding to a task. For this patient, compared with the labeled ground truth, the clustering results were satisfactory; see Figure 3A. There were minor discrepancies, such as one false clustering during picture naming and one during W/NW for spectral clustering, as well as one false labeling during W/NW for BIRCH clustering. To further investigate task-related differences, we projected the functional connectivity features onto two components using the t-SNE technique and visualized the three tasks in this low-dimensional space. As shown in Figure 3B, this representation reveals a clearer separation between task groups that was not evident in the original high-dimensional feature space. To visually validate that the clustering accuracy aligns with this observed separation, we also projected the results from both the spectral and BIRCH clustering algorithms onto the same t-SNE space, as shown in Figure 3C,D, respectively.

Then, we summarized the performance of BIRCH clustering for 12 patients using five metrics: normalized mutual info (MI), Fowlkes–Mallows (FM), adjusted Rand (AR), accuracy, and the balanced accuracy score (BAS) (Figure 3B). The distribution of metrics values exhibited a wide range, influenced by the number and locations of electrodes (see Figure 3C). Additionally, we assessed the precision of BIRCH clustering across different tasks, revealing that it is more associated with individual patients (numbers and locations of electrodes), rather than specific tasks (see Figure 3D).

### 3.3. Identifiable Cognitive Tasks from Functional Connectivity

**Figure 4:**
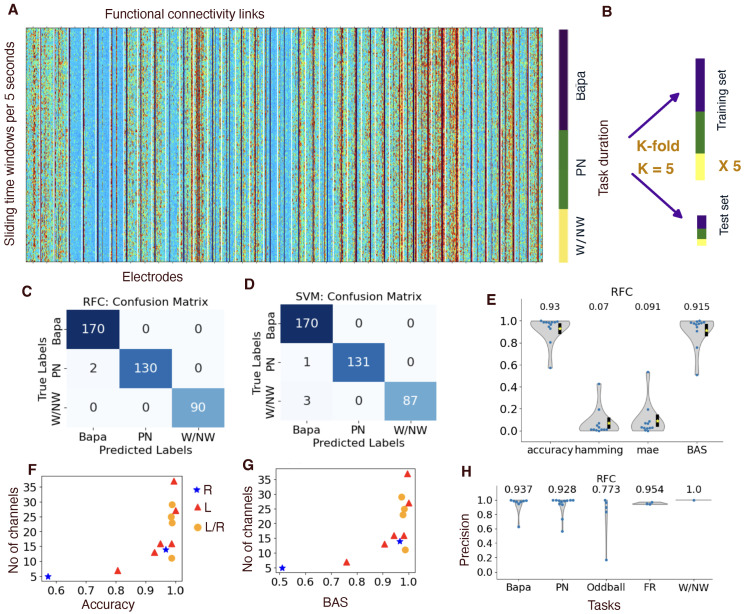
Identifiable cognitive tasks from functional connectivity. (**A**) left: the functional connectivity links between electrode leads (x-axis) and sliding time windows (y-axis). Right: the time windows during different tasks: ba/pa, picture naming (PN), and W/NW. These results are from the same patient as Figure 1 and Figure 3. (**B**) We employed Stratified K-fold cross-validation (with K=5) to ensure that each fold maintained a similar distribution of task labels. In each fold, 80% of the data was used for training and 20% for testing. (**C**) Confusion matrices from the random forest classifier (RFC), aggregating predictions across all five folds. (**D**) Confusion matrices from the support vector machine (SVM). (**E**) Performance of RFC classifier to predict a task from a given functional connectivity through four metrics: accuracy, the Hamming loss, the mean absolute error (mae), and the balanced accuracy score (BAS). (**F**,**G**) Number of electrode channels in a function of accuracy and BAS from results of (**F**). (**H**) Precision from RFC algorithm in terms of different tasks.

Can we determine the cognitive tasks associated with a given functional connectivity through training? For each patient, we applied Stratified K-Fold cross-validation (with K=5) to ensure that each fold maintained a similar distribution of task labels. In each fold, 80% of the data was used for training and 20% for testing Figure 4A,B. We employed a random forest classifier (RFC) and a support vector machine (SVM) to learn the identification of cognitive tasks from functional connectivity. In the case of the example patient, the confusion matrix revealed two instances of false labeling for RFC (Figure 4C), but one instance of false labeling of ba/pa as picture naming and three instances of false labeling of Bapa for W/NW (Figure 4D).

We then summarized the performance of RFC for 12 patients using four metrics: accuracy, Hamming loss, and mean absolute error (mae) and the balanced accuracy score (BAS) (see Figure 4E). Both accuracy and the balanced accuracy score (BAS) approached 1.0 when the number of electrode channels exceeded 16. The worst-case performance occurred in a patient with only five electrodes, all located within the right hemisphere. (See Figure 4F,G.) Furthermore, we evaluated the precision of RFC across different tasks, revealing that Bapa and PN are easier to correctly identify compared to oddball and FR (see Figure 4H).

### 3.4. Links of Interest

We have demonstrated functional connectivity at the network level for individual patients. Now, we focus on the roles of brain regions in functional connectivity (i.e., nodes in the network). We obtained the functional connectivity among the brain regions by mapping the maximal link weights among the electrodes between each pair of brain regions (see Figure 1E).

**Figure 5:**
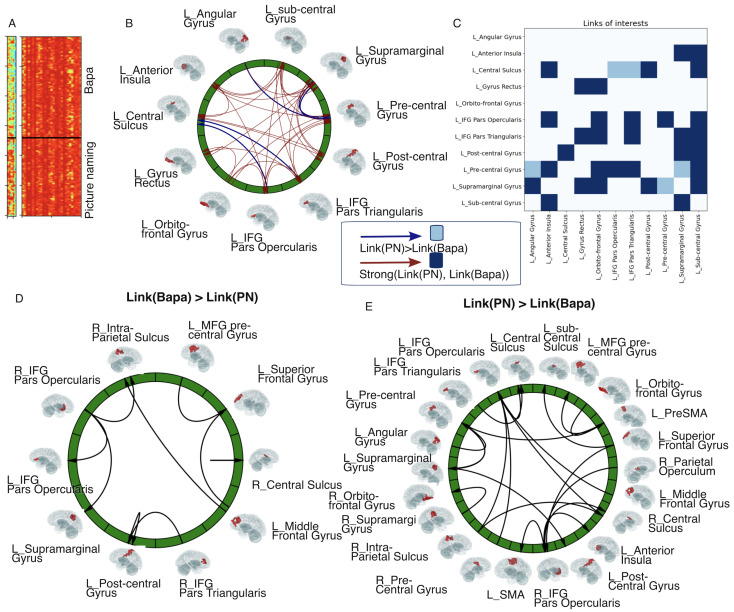
Differential links of interest between picture-naming and Bapa tasks. (**A**–**C**) show results from Patient 01, while (**D**,**E**) show results from eight patients who performed both tasks. (**A**) Two groups of links: on the left, links with weak strength during the Bapa task but much more strength during the picture-naming task. These links are indicated with blue arrows in (**B**) and light blue blocks in (**C**). On the right side of (**A**), links that were strong during both tasks are shown. These links are represented with red arrows in (**B**) and dark blue blocks in (**C**). The black bars in (**A**) separate the two tasks. (**D**) Links with greater strength during the Bapa task compared to picture naming. (**E**) Links with greater strength during the picture-naming task than during the Bapa task.

The first question at the brain region level is as follows: what are the links of interest when comparing picture naming and Bapa tasks? Taking Patient 01 as an example, we grouped the links of interest into two classes: (1) links that are strong in both tasks and (2) links that are stronger during picture naming compared to the Bapa task (see Figure 5A–C). We used the Wasserstein distance, a similarity metric between two distributions of links, to classify these groups [53]. For this patient, we identified four links that were stronger during picture naming—three involving the left precentral gyrus and two involving the left central sulcus and the left supramarginal gyrus. No links were stronger during the Bapa task compared to picture naming.

Expanding to the group results of eight patients who performed both tasks, we identified nine links that were stronger during the Bapa task compared to picture naming, with the right IFG pars opercularis serving as the source projecting to other regions (Figure 5D). In contrast, 20 links were stronger during picture naming compared to Bapa, with the right IFG pars opercularis acting as a sink for incoming connections. The left IFG pars opercularis also functioned as an additional sink (Figure 5E).

### 3.5. Connectivity Profiles of Brain Regions During Cognitive Tasks

Centrality indices are essential for identifying important nodes within a network, yet the concept of ‘importance’ can vary, leading to diverse centrality definitions. In this study, we focus on seven metrics. Degree centrality, for instance, quantifies the number of ties linking a node, with consideration given to both in-degree and out-degree in our directed functional connectivity. Closeness centrality measures a node’s proximity to all others in the graph, emphasizing nodes with shorter paths to the rest. Betweenness centrality, on the other hand, highlights nodes crucial for communication by quantifying how often they lie on the shortest paths between other nodes. Katz centrality, a generalization of eigenvector centrality, calculates a node’s centrality based on its neighbors’, considering both incoming and outgoing edges. These metrics collectively offer insights into the network’s structure and dynamics, called connectivity profiles of regions, guiding our analysis of brain communication patterns. For example, we plot the connectivity profiles of a brain region from the example patient’s data in Figure 1F.

**Figure 6:**
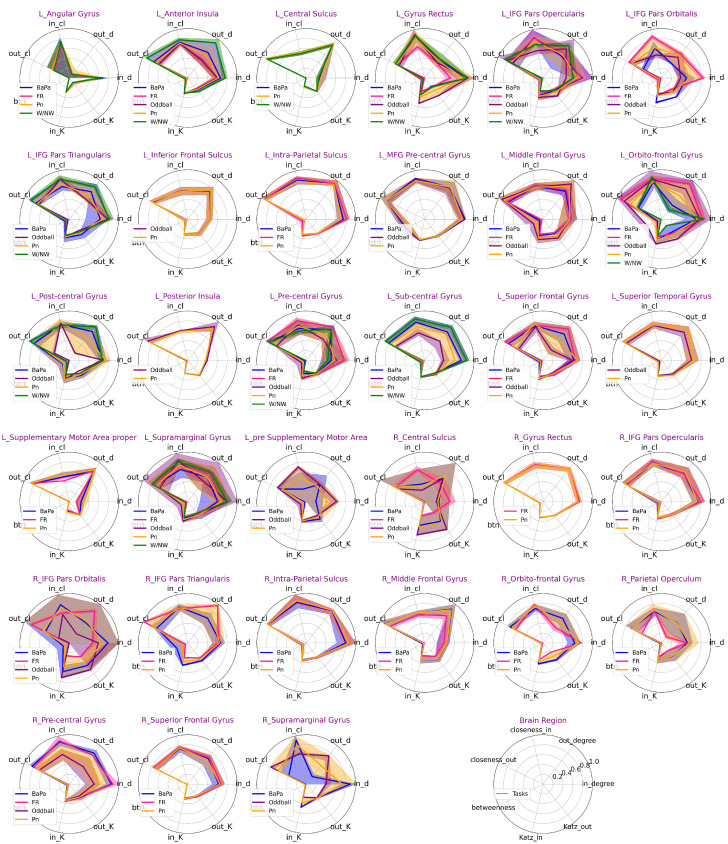
Statistical connectivity profiles of 33 brain regions in 12 patients during five cognitive tasks. For each brain region, connectivity profiles consist of seven metrics, in-degree (in_d), out-degree (out_d), closeness-in (in_cl), closeness-out (out_cl), betweenness (btn), Katz-in (in_K), and Katz-out (out_K). The ranges of all radial axes are standardized to [0, 1] for clear comparison. Lines in different colors represent the mean values of the distribution of connectivity profiles across different tasks. The shaded areas indicate the interquartile range (25th to 75th percentiles). The number of tasks each region is involved in varies, as shown in the legends.

Since the implantation of electrodes varies for each patient, our analysis holds meaning primarily at the individual level. Now, we can include group analyses across the different patients with recordings from the same brain regions. In this study, we presented the connectivity profiles of 33 brain regions in 12 patients across five tasks (see Figure 6). These connectivity profiles illustrate the roles of brain regions within the connectivity network during specific tasks. For instance, the left orbito-frontal gyrus exhibits strong roles during the oddball task, as indicated by high-profile metric values. However, the out-degree metric shows wide variability across tasks, ranging from high to low values during face recognition, oddball, picture-naming, Bapa, and N/NW tasks.

**Figure A1:**
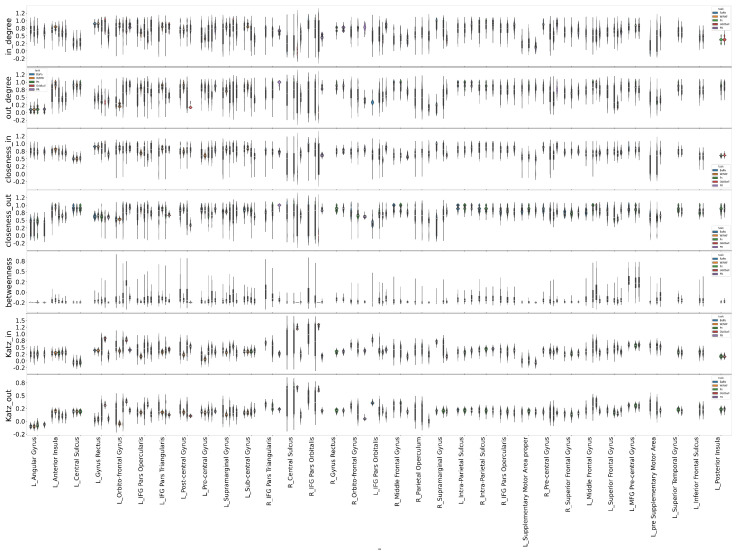
Distribution of seven connectivity metrics across 33 brain regions in five Cognitive Tasks in violin plots. Each row represents a different metric, showing the distribution of in-degree, out-degree, closeness-in, closeness-out, betweenness, Katz-in, and Katz-out.

When the roles of brain regions within their respective networks are compared across different tasks, reference to Appendix A Figure A1 is pertinent. For instance, a comparison between the left anterior insula and the left gyrus rectus reveals differences. The left anterior insula consistently exhibits a stronger out-degree across most tasks, while the left gyrus rectus tends to show a stronger in-degree. Additionally, when their neighboring regions are considered, the left anterior insula demonstrates higher Katz-out values in four tasks but lower values during the W/NW task.

### 3.6. Spatial Connectivity Features for Different Tasks

**Figure A2:**
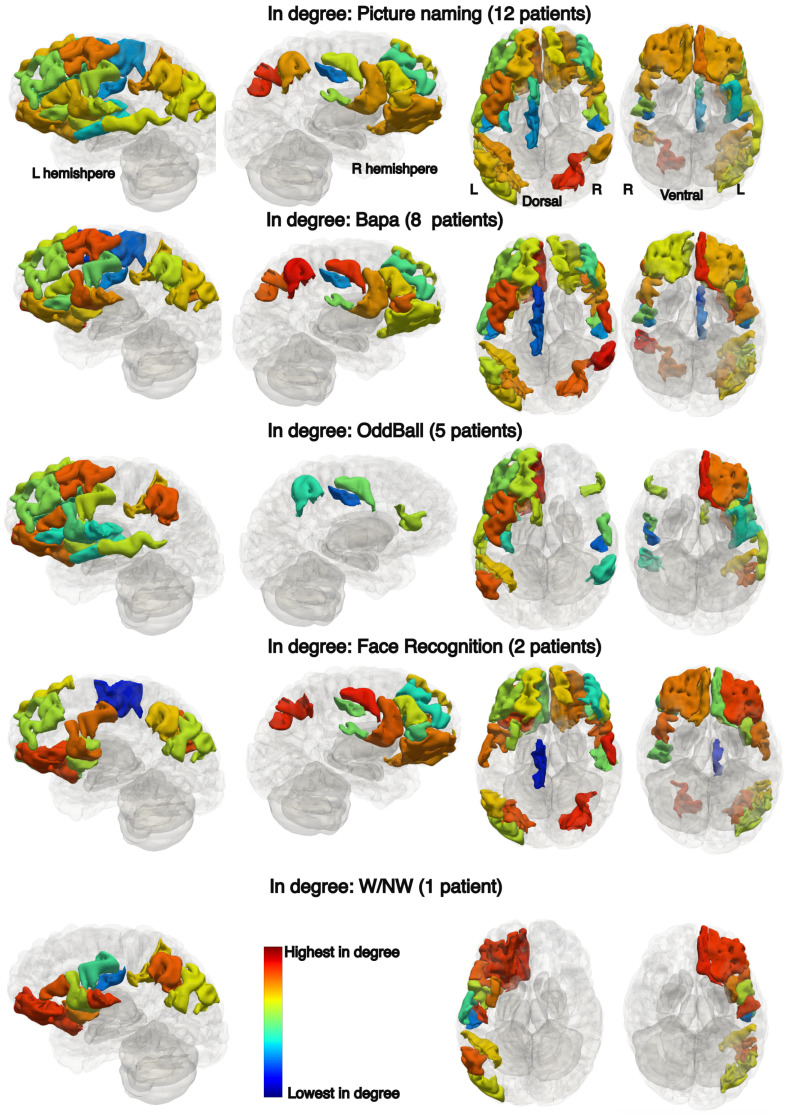
In-degree of 33 brain regions in the functional connectivity during five cognitive tasks in 3D spatial maps.

**Figure A3:**
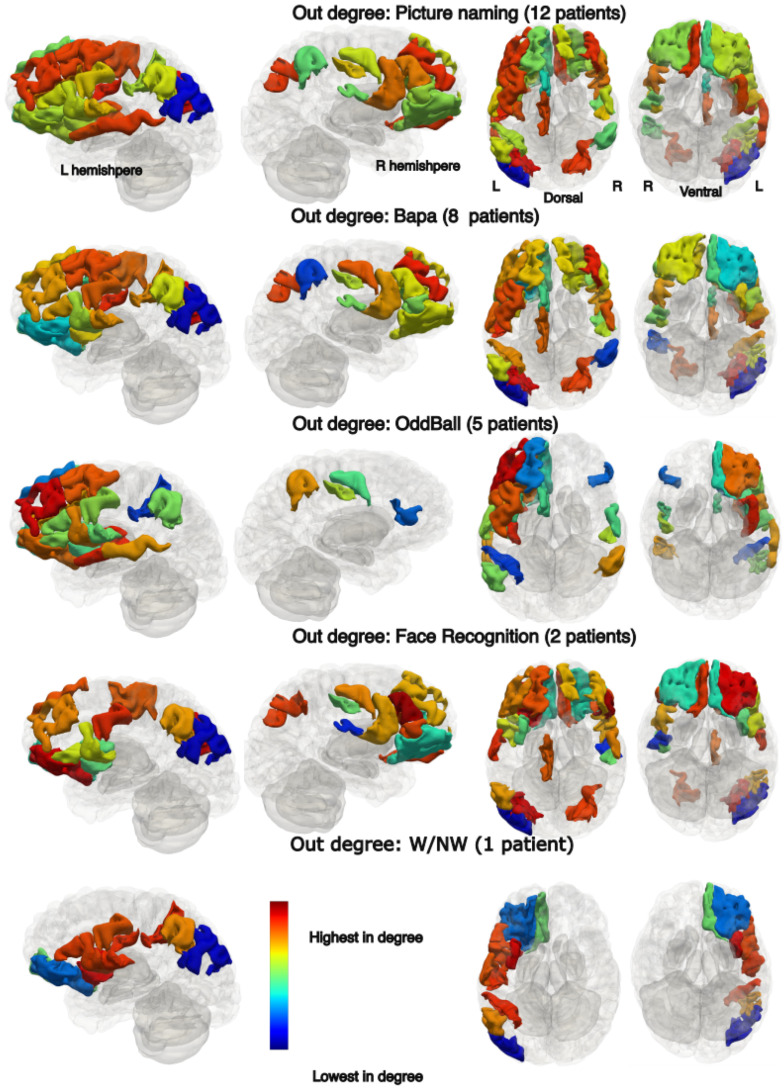
Out-degree of 33 brain regions in the functional connectivity of during five cognitive tasks in 3D spatial maps.

**Figure A4:**
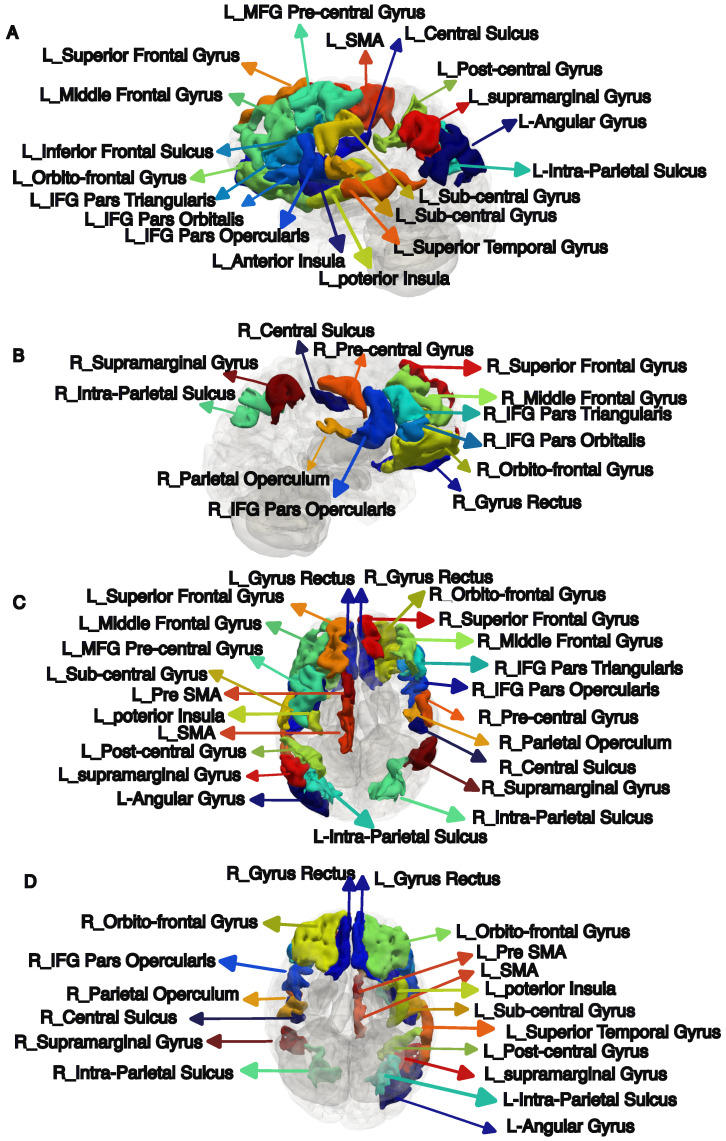
Labels of 33 brain regions in 3D maps from four views: (**A**) left hemisphere in lateral view; (**B**) right hemisphere in lateral view; (**C**) dorsal view; (**D**) ventral view.

We also illustrated the spatial distribution of in-degree and out-degree across various tasks (see Figure A2, Figure A3 and Figure A4). In the left hemisphere, the MFG pre-central gyrus exhibited high in-degree centrality across three tasks: picture naming, ba/pa, and oddball. This region is not involved in face recognition and where the left orbito-frontal gyrus demonstrates high in-degree centrality. While the spatial distribution patterns of in-degree in the left hemisphere appear visually similar across tasks, there is a greater diversity in the patterns observed for out-degree centrality.

**Figure 7:**
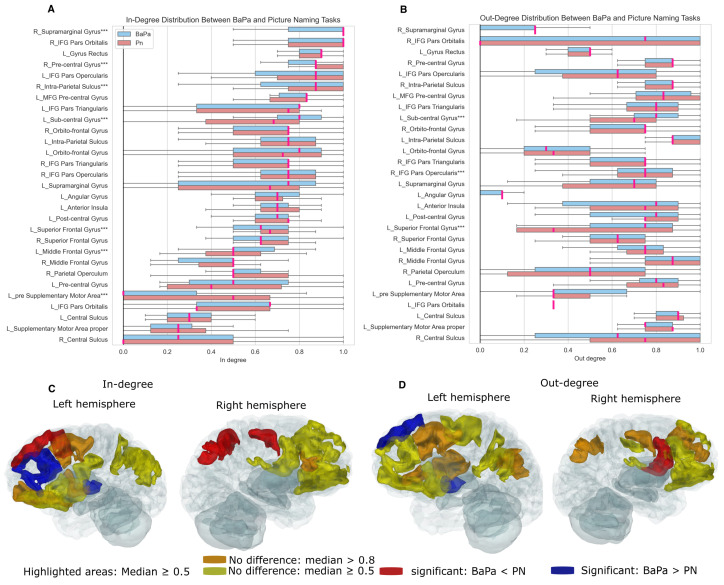
Comparison of in-degree and out-degree between the ba/pa and picture-naming tasks in eight patients. (**A**,**B**) In-degree and out-degree distributions for the ba/pa and picture-naming tasks, respectively. In-degree and out-degree values are normalized from 0 to 1. Picture naming is represented in light red and ba/pa in light blue, with deep pink indicating the median of the distributions. The areas marked with *** indicate a significant difference between the two tasks according to the Mann–Whitney U test. (**C**) Highlighted areas based on their median in-degree (≥0.5) in either or both tasks. A Mann-Whitney U test was used to determine whether the in-degree of one task was significantly greater than the other. If the in-degree of ba/pa is significantly larger than that of picture naming (*p*-value < 0.001), the areas are shown in blue; if the opposite is true, the areas are shown in red. If there is no significant difference, the areas are highlighted in yellow (median in-degree (≥0.5)) and orange (median in-degree >0.8). (**D**) The same as (**C**) but for out-degree.

We zoomed in on the brain regions whose in-degree and out-degree were available during the ba/pa and picture-naming tasks in eight patients who performed both tasks. Figure 7A,B presents the in-degree and out-degree distributions across 29 brain regions. We identified 26 regions with a median in-degree of ≥0.5 shown in Figure 7C. Among them, five regions (right supramarginal gyrus, right precentral gyrus, right intra-parietal sulcus, left superior frontal gyrus, and left pre-supplementary motor area) showed a significantly higher in-degree during picture naming, whereas the left subcentral gyrus and left middle frontal gyrus exhibited a significantly higher in-degree during the ba/pa task. Additionally, four regions (right IFG pars orbitalis, left gyrus rectus, left IFG pars opercularis, and left MFG pre-central gyrus) did not show significant differences between tasks but had consistently high in-degree values (>0.8). For out-degree, 24 brain areas had a median out-degree of ≥0.5 shown in Figure 7D. The right IFG pars opercularis displayed a significantly higher out-degree during picture naming, while the left subcentral gyrus and the left superior frontal gyrus exhibited greater source activity during the ba/pa task. Notably, eight regions (right precentral gyrus, right intra-parietal sulcus, left MFG precentral gyrus, left IFG pars triangularis, left intra-parietal sulcus, right middle frontal gyrus, left precentral gyrus, and left central sulcus) played a crucial role as source regions in both tasks.

**Figure 8:**
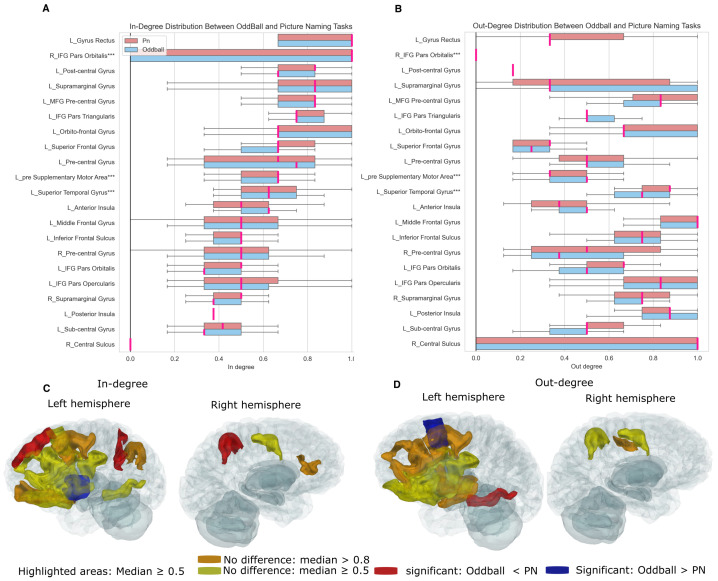
Comparison of in-degree and out-degree between the oddball and picture-naming tasks. (**A**,**B**) In-degree and out-degree distributions for the oddball and picture-naming tasks, respectively. In-degree and out-degree values are normalized from 0 to 1. Picture naming is represented in light red and oddball in light blue, with deep pink indicating the median of the distributions. The areas marked with *** indicate a significant difference between the two tasks according to the Mann–Whitney U test. (**C**) Highlighted areas based on their median in-degree (≥0.5) in either or both tasks. A Mann-Whitney U test was used to determine whether the in-degree of one task was significantly greater than the other. If the in-degree of oddball is significantly larger than that of picture naming (*p*-value < 0.001), the areas are shown in blue; if the opposite is true, the areas are shown in red. If there is no significant difference, the areas are highlighted in yellow (median in-degree (≥0.5)) and orange (median in-degree > 0.8). (**D**) The same as (**C**) but for out-degree.

Next, we compared both in-degree and out-degree between the oddball and picture-naming tasks. Figure 8A,B presents the in-degree and out-degree distributions across 21 brain regions from five patients. We identified 18 regions with a median in-degree of ≥0.5 shown in Figure 8C. Among them, three regions (left postcentral gyrus, left superior frontal gyrus, and right supramarginal gyrus) showed a significantly higher in-degree during picture naming, whereas the left anterior insula exhibited a significantly higher in-degree during the oddball task. Additionally, four regions (left gyrus rectus, right IFG pars orbitalis, left supramarginal gyrus, and left MFG precentral gyrus) did not show significant differences between tasks but had consistently high in-degree values (>0.8). For out-degree, 16 brain areas had a median out-degree of ≥0.5 shown in Figure 8D. The left superior temporal gyrus displayed a significantly higher out-degree during picture naming, while the left pre-supplementary motor area exhibited greater source activity during the oddball task. Notably, five regions (left MFG precentral gyrus, left middle frontal gyrus, left IFG pars opercularis, left posterior insula, and right central sulcus) played a crucial role as source regions in both tasks.

## 4. Discussion

Functional connectivity refers to the statistical dependence of neural activity patterns across brain regions over time [1,54]. Functional magnetic resonance imaging (fMRI; [55,56]) is the most commonly used modality in functional connectivity studies, offering whole-brain coverage but limited temporal resolution. In contrast, stereo-electroencephalography (SEEG) provides millisecond-level temporal resolution, aligning well with the fast dynamics of language, speech, and other cognitive functions. SEEG captures rich temporal and dynamic information and offers spatial resolution sufficient to examine sub-regional differences within key network areas. Its primary limitation is restricted spatial coverage due to its invasive nature. However, when interpreted within an appropriate cognitive framework, SEEG can reveal the dynamic interactions between cortical sites that underlie cognitive task processing. These interactions reflect the coordinated activity of distributed cortical regions engaged in specific components of task execution.

The present study aimed to identify functional cortico-cortical connections engaged during both language-related and non-verbal tasks and to assess whether spatial connectivity features can predict ongoing cognitive states across these tasks. To this end, we investigated the functional connectivity of the fronto-parietal network across different tasks, each engaging this network to varying degrees. The findings provide strong empirical support for previous studies based on fMRI data, which suggested that task performance reflects an interplay between intrinsic brain networks and task-specific reconfigurations [2,57]. While intrinsic networks account for commonalities across tasks, task-specific configurations highlight their functional distinctions.

To our knowledge, the comparison of dynamic functional connectivity between verbal and non-verbal tasks that tap into different levels of cognitive processing has not been previously conducted. Due to the limited electrode coverage in our patient cohort—which introduces potential bias, as not all patients performed all tasks—we focused our comparison on in-degree and out-degree centrality during three key tasks: the picture-naming task (considered the reference task; verbal), ba/pa (passive syllable listening; verbal), and the oddball task (non-verbal). The spatial distribution of in-degree and out-degree highlights the high number of connections that the MFG displays during all three tasks. The high involvement of MFG, part of the dorsolateral prefrontal cortex, illustrates its crucial role in cognitive control and attentional modulation. This finding aligns with previous work demonstrating the MFG’s involvement in higher-order speech processing using electrocorticography (ECoG) [58], as well as in phoneme discrimination and deviance detection during oddball tasks [16].

The analysis of the spatial distribution pattern of in-degree revealed a broader set of regions showing similarity across tasks, compared to the distribution of out-degree. We observed notable in-degree overlap within the fronto-parietal network between the picture naming and ba/pa tasks, suggesting a shared functional-anatomical network. However, this similarity also highlights the SFG as a key region with increased involvement during picture naming. While the SFG is not traditionally considered a core language network, it contributes to the cognitive control aspects of naming—for instance, in selecting the appropriate word while suppressing competing alternatives. It also contributes to maintaining the visual representation of the picture in memory during word retrieval and articulation. Furthermore, the SFG supports attentional processes by directing focus to relevant features of the image to ensure efficient lexical access [59,60]. These findings contribute to a more nuanced understanding of brain regions that serve as convergence points for multiple cognitive processes.

The comparison between the picture naming and oddball tasks revealed differential modulation of brain regions engaged in both tasks. The visual oddball paradigm is commonly used to assess the brain’s ability to detect and orient attention toward a target stimulus. Early electrophysiological studies identified the P300 component of the event-related potential (ERP), a positive peak occurring approximately 300 ms after stimulus presentation, in response to rare target stimuli presented among frequent standard stimuli [61]. The P300 is considered a physiological marker of information processing, reflecting stimulus perception, information integration, attentional resource allocation, and decision-making processes. This response is generated by large-scale networks involving both frontal and parietal regions and their interactions [62]. More recently, functional magnetic resonance imaging (fMRI) studies have further delineated these networks, identifying the preferential involvement of dorsal and ventral frontoparietal systems in oddball processing (for review, see [63]). While many of the regions activated during the oddball task are also engaged during picture naming, we observed that the STG acts as a driver node specifically during the picture naming task, indicating its stronger role in linguistic processing compared to attentional shifts required in the oddball task.

Functional connectivity and functional connectivity dynamics are becoming key data features for inferring personalized parameters in virtual brain twins [64,65]. Virtual brain twins are personalized whole-brain network models based on an individual’s brain data, designed for both scientific and clinical applications [64]. This concept is emerging as a promising tool for advancing precision medicine and helping scientists understand underlying mechanisms. Current research focuses on resting-state networks, but task-related networks could provide richer and more informative data features, leading to significant advances in neuroscience. The results of empirical data play two important roles in virtual brain twins: (1) they provide foundational knowledge for understanding tasks and brain states, which aids in selecting and designing appropriate models and (2) they serve as data features for inferring individual-specific parameters. Additionally, the methodology presented in this paper offers tools for analyzing functional connectivity dynamics and network profiles, contributing to the development of virtual brain twins based on task-related data.

One limitation of this study is that we analyzed data from a small cohort of 12 patients, with a sampling bias in the coverage of the same brain structures across patients. However, applying the same methodology and data analysis to a larger cohort with more extensive coverage of the same structures would allow us to obtain more stable statistical results and enable comparisons of anatomo-functional networks across cognitive tasks and resting-state conditions.

## Data Availability

The patients’ raw datasets cannot be made publicly available due to data protection concerns regarding potentially identifying and sensitive patient information. Interested researchers may access the datasets by contacting the corresponding authors.

## Appendix A

**Table A1:** Participant characteristics and task assignments.

Case	Age	Sex	Handedness	Language Dominance (Criterion)	Epilepsy	Onset (Years)	Tasks
P01	41	F	Right	Left (ictal aphasia & fMRI)	Temporo-parietal	12	PN/Ba-pa
P02	45	M	Right	NA (Not available)	R MTL	0.75	PN/FR
P03	34	F	Right	NA	R LTL	29	PN/BaPa/OB
P04	42	F	Left	Bilateral (Wada)	R MTL	33	PN/BaPa
P05	28	F	Right	Left (stimulation)	L MTL	21	PN/BaPa
P06	61	F	Right	Left (stimulation)	L Temporo-Perisylvian	50	PN/BaPa/FR
P07	53	F	Right	Left	L MTL	35	PN/OB
P08	21	M	Right	NA	R FT	13	PN/BaPa
P09	48	F	Left	Left	L MTL	42	PN/BaPa/OB
P10	27	M	Right	Left (ictal aphasia)	Bi post Insula-temporal operculum	8	PN/BaPa
P11	40	F	Right	Left (Wada test)	L MTL	17	PN/OB
P12	25	M	Right	Left (Wada test)	L IFG & OF	7	PN/OB
